# Metabolomic Insights Into Endophyte-Derived Bioactive Compounds

**DOI:** 10.3389/fmicb.2022.835931

**Published:** 2022-03-02

**Authors:** Sushma Mishra, Priyanka, Shilpi Sharma

**Affiliations:** ^1^Plant Biotechnology Laboratory, Dayalbagh Educational Institute, Deemed-to-be-University, Agra, India; ^2^Department of Biochemical Engineering and Biotechnology, Indian Institute of Technology Delhi, New Delhi, India

**Keywords:** metabolomics, bioprospecting, bioactive compounds, natural products, endophytes

## Abstract

Among the various plant-associated microbiota, endophytes (the microbial communities inhabiting plant endosphere without causing disease symptoms) exhibit the most intimate and specific association with host plants. Endophytic microbes influence various aspects of plant responses (such as increasing availability of nutrients, tolerance against biotic and abiotic stresses, etc.) by modulating the primary and secondary metabolism of the host. Besides, endophytic microbes produce a diverse array of bioactive compounds, which have potential applications in the pharmaceutical, food, and cosmetic industries. Further, there is sufficient evidence for endophyte-derived plant metabolites, which could be pursued as alternative sources of commercially important plant metabolites. The field of bioprospecting, the discovery of novel chemistries, and endophyte-mediated production of plant metabolites have witnessed a boom with the advent of *omics* technologies (especially metabolomics) in endophyte research. The high throughput study of small metabolites at a particular timepoint or tissue forms the core of metabolomics. Being downstream to transcriptome and proteome, the metabolome provides the most direct reflection of the phenotype of an organism. The contribution of plant and microbial metabolomics for answering fundamental questions of plant-endophyte interaction, such as the effect of endophyte inoculation on plant metabolome, composition of metabolites on the impact of environmental stressors (biotic and abiotic), etc., have also been discussed.

## Introduction

Bioactive compounds are metabolites that affect one or more metabolic processes, resulting in the improvement of health conditions (Angiolillo et al., 2015). Chemically, bioactive compounds are a heterogeneous group, including peptides, saccharides, phenolics, terpenes, and alkaloids. They may have widespread biological effects such as antimicrobial, antioxidant, anticancerous, etc., and hence, have great applications in the pharmaceutical, cosmetic, and food industries. The increase in population and emergence of new virulent strains and drug-resistant pathogens necessitates both, increasing the production, as well as discovering novel bioactive compounds (Gakuubi et al., 2021). Plant metabolites are a rich source of pharmacologically important compounds that aid in drug discovery and contribute to ∼80% of all synthetic drugs (Bauer et al., 2014). However, the traditional approach of extracting secondary metabolites (SM) from medicinal plants, suffers from several limitations including seasonal dependence, a gap in demand and supply, loss of biodiversity, the endangered status of many plant species, and increased costs. Here, it needs to be mentioned that plants are not always the sole producers of their SMs; endophytes (the microorganisms inhabiting the endosphere of plants without causing disease symptoms) have been found to produce host (plant) metabolites including taxol, vincristine, vinblastine, camptothecin, hypericin, etc. Endophytes are associated with almost every higher plant studied till date, inhabiting both above ground and below ground plant parts (Rigobelo and Baron, 2021). The significance of endophytes in determining the host plant’s overall fitness has been long established (Gouda et al., 2016). Besides their role in nutrient management (Johnston-Monje and Raizada, 2011; Cipriano et al., 2021; García-Latorre et al., 2021), their contribution to alleviation of stresses is significant (Verma et al., 2021; White et al., 2021). This is majorly governed through production of bioactive metabolites. The cross talk between plant and endophyte, which is affected by various abiotic and biotic factors, regulates the production of bioactive metabolites (Rai et al., 2021). This has been extensively reviewed by Mishra et al. (Mishra et al., 2021a,c).

## Bioactive Compounds From Endophytes

The endophytic microbiota inhabit diverse niches within plants, interacting with numerous co-occurring microbes, and displaying different lifestyles (mutualistic, commensal, and parasitic) (Mishra et al., 2021a). The ability of endophytes, especially fungi and actinomycetes, to produce a plethora of bioactive compounds (Supplementary Table 1) could be attributed to various reasons: chemical defense of plants due to their long term association (reported from fossil plants as well), interaction with co-occurring microbes, and protection of their niche (within plant endosphere) against attacking pathogen species (Krings et al., 2007; Kusari et al., 2014a; Mishra et al., 2021a). Several endophyte-mediated/assisted plant responses are mediated through the production of a myriad of compounds by these microbial communities (Maciá-Vicente et al., 2018).

The diversity of endophytes, and thereby of the bioactive compounds produced by them, is dependent on a range of factors including plant species and parts (Wang et al., 2019; Guevara-Araya et al., 2020; Harrison and Griffin, 2020), and environmental factors (Giauque and Hawkes, 2013). Amongst environmental factors, cultivation history (Correa-Galeote et al., 2018), and climate and season (Zimmerman and Vitousek, 2012; Hosseyni-Moghaddam and Soltani, 2014; Wang et al., 2019; Oita et al., 2021) have been established as important criteria in shaping the endophytic communities in plants.

Most of the protocols for discovery of bioactive compounds from microbial culture involve axenic cultivation (either submerged or solid-state fermentation) for 2–4 weeks, followed by bioassay-based screening (eg. anticancerous, antimicrobial, etc.) to guide the subsequent purification process. Alternatively, PCR-based screening for biosynthetic gene clusters (BGCs) such as non-ribosomal peptide synthetase (NRPS) and polyketide synthase (PKS), could provide crucial leads for identifying promising endophytic isolates (Mohamad et al., 2018). Further, approaches such as co-cultivation with host plant extract or co-occurring microbes, the addition of epigenetic modifiers, and heterologous expression have been used to activate the cryptic BGCs and induce the expression of additional metabolites (Toghueo et al., 2020). These procedures involving optimization of fermentation conditions through standardization of physico-chemical parameters and addition of elicitors are time-consuming and cumbersome. The application of metabolomics has facilitated the rapid and (almost) complete screening for metabolites, identification of novel compounds and metabolite markers in plant and endophyte extracts. This review focuses on the application of metabolomic techniques in endophyte research for the identification of economically important compounds (including plant metabolites), the discovery of novel metabolites, and offering insights into the functional role of endophytes in plant holobiont, but before that, a brief account of the technique is presented.

## Metabolomics Approach: a Brief Overview

With the advent of *omics* technologies (mainly metagenomics, metatranscriptomics, metaproteomics, and microbial metabolomics) in plant microbiome research, there has been a leap in our understanding of the composition and abundance of plant-associated microbial diversity, and the role of the plant microbiome in affecting host’s responses (Kaul et al., 2016). While metagenomic analyses of plant samples provide a snapshot of the abundance and functional potential of the plant-associated microbiota (Sessitsch et al., 2012; Mishra et al., 2021b), the metatranscriptomic and metaproteomic approaches reveal the “expressed” (RNA and protein components, respectively) fraction of the microbial genome (Liao et al., 2019; Guo et al., 2021). However, since metabolites constitute the last step of gene expression, the high throughput quantitative analysis of metabolites would provide the ultimate and most authentic representation of the physiological state of an organism. Broadly, metabolite analysis can be performed at various levels: (i) target analysis (precise measurement of the concentration of a limited number of known metabolites), (ii) metabolite profiling (non-targeted measurement of metabolite levels), (iii) metabolic fingerprinting (generates total profile or fingerprint, representing a snapshot of the metabolism, without precise quantification of metabolites), and (iv) metabolomics (detection and quantification of thousands of metabolites, defining the phenotype of the biological system) (Shulaev et al., 2008; Porzel et al., 2014).

Metabolomics is the high throughput study of small metabolites (Mr ≤ 1 kDa) present in a sample (Nalbantoglu, 2019). Such metabolites, including peptides, sugars, fatty acids, organic acids, vitamins, etc., could represent intermediates or end-products of metabolic pathways, or those that are formed due to the impact of environmental factors or biotic stressors (Castro-Moretti et al., 2020). The analysis of plant and endophyte metabolomes provide a direct insight into the contribution of microbiome toward host plant phenotype, as well as the impact of environment on plant-microbe interactions, thereby serving as sensitive and accurate markers. Moreover, the ability to scale up and analyze hundreds of samples simultaneously has brought a revolution in the field of natural product discovery and bioprospecting of endophytes to produce diverse compounds. The application of microbial metabolomics enables the complete analysis of crude extracts as well as the identification of marker metabolites (associated with specific bioactivities), before undergoing tedious and laborious downstream processes (Mohamed et al., 2020).

**FIGURE 1:**
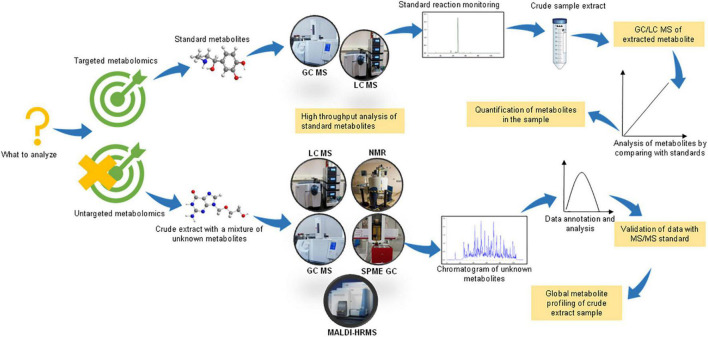
Experimental design for performing untargeted and targeted metabolomic studies. Figure comprised of images taken from google images (source license ‘CC0’).

Based on the objective of the study, there could be two basic experimental designs for conducting a metabolomic analysis, viz. untargeted/non-targeted and targeted metabolomics (Figure 1). As the name suggests, non-targeted metabolomics involves a comprehensive analysis of numerous small metabolites in the sample. The sample preparation includes the extraction of metabolites using two or more solvents to increase the range of detection of chemically diverse metabolites. This is a hypothesis-generating method and enables the discovery of novel compounds. On the other hand, targeted metabolomics, also known as directed metabolomics, focuses on the quantitative analysis of a specific class of metabolites (∼50 metabolites), usually by tandem MS/MS method against a standard. It is often performed for validating the results of untargeted metabolomics (Tebani et al., 2018). Therefore, the non-targeted and targeted metabolomic approaches could be summarized as “untargeted-discovery-global” and “targeted-validation-tandem, respectively (Nalbantoglu, 2019).

There are two analytical platforms for metabolite detection, viz. nuclear magnetic resonance (NMR) and mass spectrometry (MS)-based; both with their own set of strengths and drawbacks (Table 1). Next, separation techniques such as gas chromatography (GC; for volatile molecules such as organic acids, fatty acids, etc.), liquid chromatography (LC; for thermo-labile substances), and capillary electrophoresis (CE; combines the advanced technology of electrophoresis and microcolumn separation) are used in conjunction with MS or NMR equipment. The readers may refer to a recent review on analytical techniques for metabolomics by Segers et al. (2019).

**TABLE 1:** Advantages and disadvantages of various metabolomic techniques.

Techniques	Advantages	Disadvantages
Nuclear magnetic resonance (NMR)	Non-destructive and rapid, Minimal sample preparation, Highly reproducible	Lack of sensitivity, Expensive instrument
Gas chromatography-mass spectrometry (GC-MS)	High sensitivity and resolution, Easy to use, Cost-effective method, Spectrum library available for unknown materials, Suitable for non–thermosensitive and volatile molecules	Not suitable for less volatile compounds, Derivatisation of samples required
Liquid chromatography-mass spectrometry (LC-MS)	High sensitivity, Suitable for less volatile metabolites, Wide range analysis, Sample derivatisation not required, Both qualitative and quantitative analysis	Lack of reproducibility with fragmentation pattern, Poor separation of different isomers
Capillary electrophoresis-mass spectrometry (CE-MS)	No special treatment required for samples preparation, Low dosage requirement, Cost effective process, Offer shorter test time, Less sample volume required	Low separation reproducibility, Not applicable for high molecular weight proteins
Ultra high performance liquid chromatography-high resolution mass spectrometry (UHPLC-HRMS)	Easy sample preparation, Faster analysis, High mass spectral resolution of metabolites, Interchangeable ion sources, High sensitivity	Formation of dimers belonging to single compound, Expensive instrumentation, Higher maintenance
Solid-phase microextraction-gas chromatography-mass spectrometry (SPME-GC-MS)	Simple and versatile, Less time consuming, No harmful solvent required	Loss in analysis and rapidity, Limited availability of commercially available fiber material
Matrix-assisted laser desorption/ionization high-resolution mass spectrometry imaging (MALDI-HRMSI)	Investigate the presence and distribution of the significant secondary metabolites. Requires limited sample preparation provides high spatial resolution	Compounds of low abundance cannot be analyzed, Structurally similar compounds cannot be analyzed

## Metabolomics for Investigation of Endophyte Metabolome

### Bioassay-Based Screening of Crude Extracts of Endophyte

Metabolomic tools have been used to compare the metabolic profile obtained from *Aspergillus aculeatus* (an endophyte isolated from *Terminalia laxiflora*) inoculated in solid and liquid media (Tawfike et al., 2017). The use of rice culture media was reported to produce a higher number of metabolites than liquid culture media. Next, using MS-based metabolomics, the group reported the production of seven metabolites with anticancerous and/or antitrypanosomal properties from the fermentation culture of *Aspergillus flocculus*, an endophyte isolated from the stem of *Markhamia platycalyx* (an Egyptian medicinal plant) (Tawfike et al., 2019). Specifically, five out of the seven metabolites detected in 30-day-old rice culture media demonstrated antagonistic activity against chronic myelogenous leukemia cell line K562, and two exhibited strong activity against the parasite *Trypanosoma brucei brucei* (causal organism of sleeping sickness).

In another report, Sebola et al. (2020) analyzed the antibacterial (against 11 pathogenic bacteria constituting both Gram-positive and Gram-negative bacteria) and anticancer activity (against glioblastoma and lung carcinoma cell lines) of ethyl acetate extracts of bacterial endophytes (*Bacillus safensis, Pseudomonas cichorii*, and *Arthrobacter pascens*) isolated from *Crinum macowanii* (commonly known as Cape coast lily; used to cleanse blood, and treat cough and kidney diseases) leaves. Further, using LC-Q-TOF-MS analysis of crude extracts of the bacterial endophytes revealed the presence of antibacterial and anticancerous compounds such as lycorine, angustine, crinamidine, and powelline. In another similar report, Nischitha and Shivanna (2021) analyzed the antimicrobial and antioxidant activities of crude extract of *Alloteropsis cimicina* (a grass species) and its endophytic fungus *Penicillium pinophilum*; the antioxidant potential of the fungal extract being more than the host extract. Metabolite profiling using Orbitrap High-Resolution (OHR) LC–MS and Fourier-transform infrared spectroscopy (FTIR) analysis identified 21 antimicrobial and 13 antioxidant compounds in the fungal extract. Some of the identified antimicrobial compounds included L-tyrosine, L-isoleucine, acetophenone, phenacetin, dextramycin, and sulfamethazine; and antioxidant compounds include citrinin, zearalenone, and bilirubin ((Nischitha and Shivanna, 2021). Interestingly, there were eight compounds (namely L-tyrosine, acetophenone, 4-methoxycinnamic acid, isophorone, asarone, zearalenone, dioctyl phthalate, and 4-hydroxycoumarin) that were common to both host and the endophyte. These reports provide encouraging evidence for plant-associated endophytes as a natural reservoir of various pharmacologically important and novel bioactive compounds.

### Metabolomics-Guided Discovery of Novel Compounds From Endophytes

**FIGURE 2:**
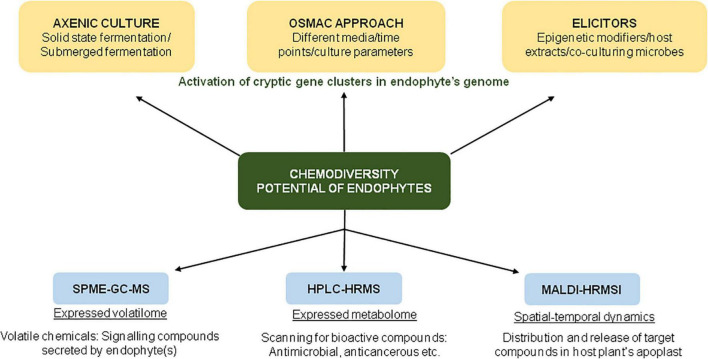
Schematic representation of various approaches for screening of bioactive compounds in endophyte cultures.

In an ambitious project, Maciá-Vicente et al. (2018) analyzed the metabolites produced by 822 root-associated fungi (from different plant species collected from various geographical locations) by untargeted ultra-performance liquid chromatography electrospray ionization tandem mass spectrometry (UPLC-ESI-MS/MS)-based approach. The study enabled comprehensive and unbiased coverage of secondary metabolites, as revealed by the detection of >17,000 compounds by UPLC-ESI-MS/MS, of which ∼9000 compounds had unique chemistries. The study was conducted with the objective to discover novel natural products, chemotaxonomic markers for fungal groups, and to facilitate bioprospecting for drug discovery (Figure 2).

*Xylaria ellisii* is a griseofulvin (antifungal activity by inhibiting microtubule assembly) producing novel fungal endophyte isolated from *Vaccinium angustifolium* (commonly known as wild blueberries). To identify novel natural products, 15 endophytic *Xylaria* strains were subjected to LC-UV/MS-based metabolomic analysis, which led to the discovery of eight new groups of compounds called ellisiiamides (proline-containing cyclic non-ribosomal pentapeptides) from ethyl acetate extract and mycelium (Ibrahim et al., 2020). The structural elucidation of these ellisiiamides was performed using NMR and LC-HRMS/MS analysis. Subsequent analysis demonstrated Ellisiiamide A to have antagonistic activity against *E. coli*, the role of other ellisiiamides is yet to be discovered.

The application of the One Strain Many Compounds (OSMAC) approach enables the researchers to explore the chemodiversity of microorganisms (Figure 2). In one such study involving an OSMAC-assisted metabolomics approach, Armin et al. (2021a) investigated the metabolic potential of *Streptomyces pulveraceus* strain ES16 (an apple-associated root endophytic bacteria) by growing it in six different media (ranging from nutrient-rich to minimal media) for a period of 3, 7, and 14 days. Using various state-of-art techniques such as SPME-GC-MS (for expressed volatilome), HPLC-HRMS*^n^* (to target the expressed metabolome), and MALDI-HRMSI (for spatial-temporal visualization of metabolites), the authors demonstrated the production of geosmin (a volatile chemical that attracts soil arthropods), mirubactin (iron-chelating siderophore) and polycyclic tetramate macrolactams (biocontrol activity against various pathogens) under stress conditions (Armin et al., 2021a). In addition to chemical OSMAC (by alteration/optimization of chemical media), biological OSMAC involving interaction with other microbial species (endophytic or pathogenic) can be used to increase the diversity of endophyte-derived bioactive compounds (Armin et al., 2021b).

### Metabolomics for Endophyte-Derived Plant Metabolites

Endophytic microbial communities represent a natural reservoir for obtaining a myriad of bioactive compounds, including host plant metabolites (Table 2). It has been proposed that due to long-term evolutionary association with plants, the symbiotic microbiota have acquired some of the host plant genes by horizontal gene transfer (Tiwari and Bae, 2020). Further, genome sequencing of microbes has demonstrated a higher potential to produce metabolites than those observed under standard laboratory conditions. Since the landmark discovery of endophyte-mediated taxol production from an endophytic fungus (*Taxomyces andreanae*) isolated from *Taxus brevifolia* (Stierle et al., 1993), several endophytic microbes have been reported to produce plant metabolites under axenic conditions, some of the reports have been discussed below.

**TABLE 2:** Studies where endophytic microbes have been reported to produce plant metabolites.

Endophyte	Host plant	Metabolite	Technique(s) used	Pharmacological activity	References
*Taxomyces andreanae*	*Taxus brevifolia*	Paclitaxel	TLC, HPLC, immunoassay, and radiolabelled precursors	Anticancer	Stierle et al., 1993
*Phialocephala fortinii*	*Podophyllum peltatum*	Podophyllotoxin	HPLC, ESI-MS	Anticancer	Eyberger et al., 2006
*Fusarium solani*	*Apodytes dimidiate*	Camptothecin 10-hydroxycamptothecin	LC-MS/MS, LC-HRMS	Anticancer	Shweta et al., 2010
*Alternaria alternate*	*Capsicum annuum*	Capsaicin	LC-ESI-MS/MS	Antitumor	Devari et al., 2014
*Colletotrichum gloeosporioides*	*Piper nigrum*	Piperine	HPLC, LC-MS	Antimicrobial, antidepressant, antiinflammatory, and anticancer	Chithra et al., 2014b
*Eupenicillium parvum*	*Sinopodophyllum hexandrum*	Kaempferol	TLC, HPLC, and NMR	Antioxidant	Huang et al., 2014
*Rhizoctonia bataticola*	*Coleus forskohlii*	Forskolin	TLC	Reduce blood pressure, antiallergic, antiinflammatory	Mir et al., 2015
*Geomyces* sp.	*Nerium indicum*	Vincamine	TLC, HPLC, and LC-MS	Cardiovascular and cerebrovascular protective	Na et al., 2016
*Fusarium solani*	*Camptotheca acuminate*	Camptothecin	TLC, HPLC, and EI-MS	Antineoplastic	Ran et al., 2017
*Aspergillus fumigates*	*Taxus* sp.	Paclitaxel	TLC, HPLC, FTIR, and NMR	Anticancerous	Kumar et al., 2019

#### Azadirachtin

Azadirachtin is an oxygenated tetranortriterpenoid found in *Azadirachta indica* (Indian neem; used to cure fever, pain, leprosy, and malaria). It is a broad spectrum insecticide, effective against pests belonging to Heminoptera, Isoptera and Orthoptera (Kilani-Morakchi et al., 2021). The axenic culture of *Eupenicillium parvum* (an endophytic fungus isolated from this plant) was obtained by inoculation in Sabouraud medium for 20 days on a rotary shaker (28°C, 200 rpm). After filtration, both the mycelial pellet and suspension culture were analyzed by LC-HRMS and compared against standard Azadirachtin. The quantitative analysis by LC-MS revealed a yield of ∼0.4 μg per 100 g dry weight of fungal mycelia, and 43 μg per liter in the spent broth for Azadirachtin A, and ∼ 0.05 μg per 100 g dry weight of fungal mycelia and 11 μg per liter in spent broth for Azadirachtin B (Kusari et al., 2012).

#### Camptothecin

Camptothecin is a pentacyclic quinoline alkaloid with antineoplastic (that prevents or halts tumor growth) properties, obtained from *Camptotheca acuminata* and *Nothapodytes nimmoniana.* Camptothecin causes DNA damage (and hence, apoptosis) by binding to and stabilizing the topoisomerase-DNA complex (Ghanbari-Movahed et al., 2021). Camptothecin and its analogs are synthesized by *Fusarium solani*, an endophytic fungus isolated from *C. acuminata* plant (Kusari et al., 2009). Interestingly, the metabolite could not be detected in the filtrate/culture medium, but accumulated in the fungal biomass, when detected using LC-HRMS and compared with the standard. In a recent report, Mohinudeen et al. (2021) reported the ability of >70% endophytic fungi isolated from *N. nimmoniana* to produce camptothecin in suspension cultures. Interestingly, one of the endophytic isolates, *Alternaria burnsii* NCIM1409, demonstrated high production of camptothecin (∼ 200 μg/g) even after 12 rounds of subculturing (without suffering from attenuation) as detected by LC–MS/MS.

#### Hypericin

The medicinal plant, *Hypericum perforatum* (commonly known as St. John’s Wort), produces hypericin (a naphthodianthrone derivative), which has several medicinal properties such as antimicrobial (bacteria, fungi, and viruses) antioxidant, anticancerous, and antidepressant. Hypericin inhibits virus replication by preventing the shedding of virus particles and its reassembly in infected cells (Dellafiora et al., 2018). The production of hypericin by endophytic fungi (*Thielavia subthermofila*) was first reported by Kusari et al. (2008); the same has been discussed in detail in the later sections of the review. Another endophytic fungi, *Epicoccum nigrum*, was also reported to produce hypericin, as detected by HPLC-UV analysis, and confirmed by LC-HRMS and LC-HRMS/MS analysis (Vigneshwari et al., 2019).

#### Piperine

Piperine is an amide alkaloid (obtained from various *Piper* species such as *Piper nigrum*, *P. longum*, and *P. chaba*), widely used in Ayurveda, Siddha, and Unani medicine systems to cure respiratory and digestive disorders (Yadav et al., 2020). Besides, piperine is also used as a bioenhancer (to increase the bioavailability of other drugs), by either blocking cytochrome P450 (the drug metabolizing enzyme) or by altering the permeability of intestinal mucosa (Tiwari et al., 2020). There have been a couple of reports on the isolation of endophytes from *P. longum* and *P. nigrum*, and confirmation of piperine production ability by HPLC and LC-MS (Verma et al., 2011; Chithra et al., 2014a,b; Mintoo et al., 2019). The piperine-producing endophytes include endophytic strains of *Colletotrichum gloeosporioides*, *Mycosphaerella* sp., and *Periconia* sp. Interestingly, Chithra et al. (2014a) could detect piperine production by the endophytic fungi *Mycosphaerella* sp. through LC-MS/MS analysis, but it could not be detected using HPLC analysis, thereby highlighting the importance of sensitivity in the screening of bioactive compounds.

#### Phenylpropanoids

The phenylpropanoid pathway is responsible for the biosynthesis of several classes of SMs such as flavonoids, isoflavonoids, lignans, and stilbenes in plants (Fraser and Chapple, 2011). Phenylpropanoids are important components of cell wall, phytoalexins (against herbivores and pathogens) and floral pigments (mediating pollination) (Deng and Lu, 2017). Interestingly, the phenylpropanoid pathway is not specific to plants; there are a couple of studies reporting the production of phenolics and flavonoids from endophytic strains namely *Aspergillus nidulans* ST22 and *A. oryzae* SX10 from *Ginkgo biloba* (Qiu et al., 2010). Recently, Lu et al. (2020) reported starvation-induced production of phenylpropanoids in *Alternaria* sp. MG1 (an endophyte isolated from *Vitis vinifera*); the pathway remains repressed under normal growth conditions. Using RT-PCR and GC-TOF-MS-based metabolomics, the authors demonstrated induction in the expression of shikimate and phenylpropanoid biosynthetic genes, as well as accumulation of metabolites such as shikimate, phenylalanine, tyrosine, and 3-(4-hydroxyphenyl)pyruvate under starvation conditions (Lu et al., 2020). The MG1 strain could convert glucose to resveratrol (a polyphenol found in grapes, having high antioxidant and anticancerous activity), thereby acting as a potential alternative source for this compound (Salehi et al., 2018). Apart from resveratrol, the metabolomic analysis also revealed the production of other metabolites such as piceatannol (stilbenoids), taxifolin (flavonoid), lignans (caffeate, sinapate, and sinapoyl aldehyde) and cuminic alcohol (used in food and cosmetic industries), thereby highlighting this endophytic strain to be a treasure house of multiple important bioactive compounds.

#### Withanolides

Withanolides are secondary metabolites made up of a steroid backbone coupled to a lactone or its derivatives found in *Withania somnifera* (commonly known as Ashwagandha or as Indian ginseng). These metabolites are widely used as memory and stamina enhancer, nerve tonic, and have cardioprotective, neuroprotective, antidiabetic, antioxidant properties, etc. (White et al., 2016). An endophytic fungus, *Taleromyces pinophilus*, isolated from leaves of *W. somnifera*, was found to produce withanolides (C28-steroidal lactones of triterpene ancestry). HPLC and ^1^H NMR analyses revealed that the endophyte produced a higher yield of withanolides (360 mg/L) than the plant itself (Sathiyabama and Parthasarathy, 2018). Another study reported an increase in withanolide content in leaves upon inoculation of *in vitro* raised *W. somnifera* plants with some endophytic strains, in comparison to non-inoculated control plants (Pandey et al., 2018). The increase in withanolide content was accompanied by modulation of expression of withanolide biosynthetic genes.

## Metabolomics for Answering Fundamental Questions

### “Actual” Source of Plant Metabolites

The secondary metabolites produced in plants could be derived either from the host, or endophytes, or a combination of both. There are a couple of examples in literature where endophytes (and not plants) have been reported to be the “actual” source of the metabolite (Mishra et al., 2021c). For example, a HPLC-HRMS-based study revealed that endophytic bacteria associated with the roots of *Putterlickia verrucosa* and *P. retrospinosa* were the original source of maytansine, an anticancerous and cytotoxic compound. The results were further substantiated by MALDI-imaging-HRMS that delineated the localization of maytansine producers in the root cortex of host plants (Kusari et al., 2014b). Another case where the endophytic community was found to be the source of the “plant metabolite” was for astins, a class of macrocyclic peptides with antitumor activity, biosynthesized by the endophytic fungi *Cyanodermella asteris* (earlier known to be produced by the host plant *Aster tataricus*) (Schafhauser et al., 2019). Likewise, the antitumor activity of the bioactive fraction of moss *Claopodium crispifolium* was attributed to *Nostoc microscopium*, an endophytic blue green alga associated with the moss plant (Spjut et al., 1988).

### Insights Into Endophytic Hypericin Biosynthesis

*Thielavia subthermophila*, an endophytic fungus (isolated from the stem of *Hypericum perforatum*, commonly known as St. John’s Wort) was found to produce hypericin (a napthodianthrone with antidepressant, antimicrobial, antiinflammatory, and antioxidant properties, as mentioned before) and emodin under submerged fermentation conditions, as detected by HPLC-MS/MS (Kusari et al., 2008). However, in contrast to its host plant, the growth kinetics and hypericin biosynthesis in the endophyte occurs irrespective of light conditions. Further, the *hyp-1* gene encoding for an enzyme that converts emodin to hypericin in *H. perforatum* cell cultures (Bais et al., 2003) could not be amplified from the endophyte genome. Also, the addition of emodin to the endophyte culture did not change the level of hypericin (or emodin), irrespective of illumination conditions. These results obtained for microbial metabolomics suggest a different mechanism of hypericin biosynthesis under axenic conditions. The endophyte has devised a different mode of regulation for hypericin, which exhibits high cytotoxicity when irradiated with visible light. The *H. perforatum* plants store this metabolite in dark glands to prevent autocytotoxicity. In the absence of such dark glands, the endophyte has “evolved” to inhabit the inner stem tissues of the host plant (from where the endophyte was isolated, and where ambient light does not reach). Therefore, microbial metabolomics has unraveled the biosynthetic and regulatory mechanism for a plant metabolite, synthesis by the endophyte, independent of its host.

### Deciphering “Taste” Differences in Contrasting Cultivars

In a recent report, a non-targeted LC-MS-based metabolomic analysis of bitter and sweet varieties of sugarcane was carried out (Huang et al., 2021). Around 20% of the total (1245) metabolites (comprising both primary and secondary metabolites) detected in stalk tissues, were differentially abundant between the two varieties. In addition, the microbial communities residing in the sugarcane varieties, as well as the soil, were investigated by 16S rRNA gene sequencing. The results revealed that the flavonoid content in sugarcane stalks and nitrogen content of the soil was correlated with the composition of soil’s microbiome. Metabolites such as lamioside, primisulfuron, 3,5-dimethylpyrazin-2-ol, sitaxentan, etc., were >30 to 60-fold higher in the bitter variety. Likewise, some of the metabolites differentially accumulating in the sweet variety included eucoside, isovitexin 2”-O-arabinoside, and vaccarin. Interestingly, the concentrations of many sugarcane metabolites including apigenin, biochanin A, hesperidin, luteolin, and trigonelline, correlated well (*r* > 0.8) with specific microbial species (Huang et al., 2021).

### Investigation of Specific Plant-Endophyte Interaction

Scherling et al. (2009) reported one of the first application of the metabolomic approach for studying specific plant-endophyte interaction using *in vitro* grown (endophyte-free) poplar plants. The inoculation of a specific endophyte, *Paenibacillus* sp. strain P22, to *in vitro* grown endophyte-free plants (the micropropagated plants regenerated from shoot meristems) caused significant changes in the metabolome (affecting levels of 32 metabolites) of inoculated vs. non-inoculated plants. The GC-TOF-MS analyses revealed distinct metabolic signatures for inoculated and non-inoculated (control) plants, indicative of remarkable changes in plants’ nitrogen assimilation. The *Paenibacillus*-inoculated poplar plants exhibited elevated asparagine (8-fold) and urea (6-fold) levels (corresponding to nitrogen fixation ability of the endophyte), and reduced levels of sugars and organic acids (intermediates of Krebs’ cycle).

## Metabolomics for Imparting Climate Resilience to Crops

Environmental metabolomics refers to the application of metabolomics for characterizing an organism’s response to environmental stresses. Another commonly used term is *eco-metabolomics*; it refers to the application of metabolomic techniques to study ecological principles, including the interaction of species with the environment and with other species (Maciá-Vicente et al., 2018). Such studies can be performed at various levels of ecological organization, namely, individual, population, community, or ecosystem. Though the review mainly focuses on the contribution of metabolomics in the discovery and identification of endophyte-derived bioactive compounds, the contribution of endophyte metabolome toward climate resilience has been briefly discussed.

### Genomics-Metabolomics Integrative Approach for Disease Suppression

The bioactive compounds or metabolites produced by endophytic microbiota are responsible for many microbiome-imparted plant phenotypes such as disease suppression. The production of metabolites by the endophytic root microbiome of sugarbeet has been effective in suppressing infection by *Rhizoctonia solani* (a soil-borne fungal root pathogen of rice, wheat, and sugarbeet), as revealed by metagenomics and network analyses (Carrión et al., 2019). The activation of NRPS-PKS biosynthetic gene clusters in bacterial endophytes leads to the synthesis of enzymes that digest fungal cell walls, as well as antifungal compounds including phenazines, polyketides, and siderophores (Carrión et al., 2019). Further, the authors designed a microbial consortium to suppress fungal root disease, using *Chitinophaga* and *Flavobacterium* spp.

In the same year, Hayden et al. (2019) applied environmental metabolomics to facilitate the discrimination of disease suppressive soils for *R. solani.* The researchers analyzed the soil profile of disease suppressive and non-suppressive soils in Australia by ^1^H NMR and LC-MS techniques, over 2 years of wheat cultivation. The application of metabolomics eliminates the lengthy screening procedures involving pot experiments and quantitative PCR under field conditions. The study revealed that disease suppressive soils had a different ^1^H NMR and LC-MS profile, mainly involving a higher content of sugar molecules, while the non-suppressive soil had more abundant lipid and terpenes. The study also identified a compound called macrocarpal (an antimicrobial secondary metabolite), as a biomarker for *R. solani* suppressive soil (Hayden et al., 2019).

It needs to be mentioned that rhizospheric microbes have been found to act as the first line of defense against fungal root pathogens in disease suppressive soils (Mendes et al., 2011). The secretion of lipopeptides (encoded by non-ribosomal peptide synthetase gene) by specific strains of *Pseudomonas* are effective in imparting plant resistance to fungal diseases (Mendes et al., 2011). These reports provide encouraging evidence for imparting disease suppression phenotype to disease-conducive soils, similar to fecal transplant in humans. However, the practical feasibility of this concept requires the inoculated microbiota to display active growth rates and tolerance to environmental fluctuations, among others.

In another report, Llorens et al. (2019) performed non-targeted metabolomics using LC-ESI full-scan mass spectrometry to analyze the metabolome of cultivated wheat plants inoculated with two endophytes, *Acremonium sclerotigenum* and *Sarocladium implicatum*, isolated from a wild ancestor *Aegilops sclerotigenum.* The metabolomic analysis revealed that the levels of metabolites involved in 2-oxocarboxilic acid metabolism and amino acid-related metabolism, mainly asparagine and glutamate, were significantly altered between the inoculated and non-inoculated plants. These amino acids are abiotic stress-responsive and involved in the production of osmolytes such as proline and polyamines (Yamamoto et al., 2015). The results indicate that endophytes improve plant performance under stress conditions by improving the physiological status of plants (Llorens et al., 2019).

## Challenges in Metabolome Mining

In metabolomics, the detection and identification of metabolites involve the comparison of experimental MS/MS spectra to library spectra (a process known as dereplication); therefore, the raw data quality and robustness of the database dictates the authenticity/success of the experiment. Further, owing to the chemical diversity of metabolites, there is no universal protocol for the detection and quantification of all the classes of metabolites. It is a costly method requiring sophisticated instrumentation and facilities, and hence, not feasible for small laboratories. The lack of reference libraries for metabolites and the availability of standards limit the applications of metabolomics. Another challenge is that the synthesis of metabolites in a natural community cannot be specifically ascribed to a specific microbial species (biological source and biosynthetic pathway). Finally, the results are sensitive to contaminants and experimental artifacts. Nevertheless, metabolomics has added another dimension to our understanding of plant-microbe interactions, and with further technological advancements, these challenges can be addressed.

## Author Contributions

SM and SS conceived the idea of the manuscript. SM wrote the manuscript. Priyanka prepared the figures and tables. All authors have read and approved the final version of the manuscript.

## Conflict of Interest

The authors declare that the research was conducted in the absence of any commercial or financial relationships that could be construed as a potential conflict of interest.

## Publisher’s Note

All claims expressed in this article are solely those of the authors and do not necessarily represent those of their affiliated organizations, or those of the publisher, the editors and the reviewers. Any product that may be evaluated in this article, or claim that may be made by its manufacturer, is not guaranteed or endorsed by the publisher.

## References

[B1] Angiolillo L. del Nobile M. A. Conte A. (2015). The extraction of bioactive compounds from food residues using microwaves. *Curr. Opin. Food Sci.* 5 93–98. 10.1016/J.COFS.2015.10.001

[B2] Armin R. Zühlke S. Mahnkopp-Dirks F. Winkelmann T. Kusari S. (2021a). Evaluation of apple root-associated endophytic *Streptomyces pulveraceus* strain ES16 by an OSMAC-assisted metabolomics approach. *Front. Sustain. Food Syst.* 5:643225. 10.3389/fsufs.2021.643225

[B3] Armin R. Zühlke S. Grunewaldt-Stöcker G. Mahnkopp-Dirks F. Kusari S. (2021b). Production of siderophores by an apple root-associated *Streptomyces ciscaucasicus* strain GS2 using chemical and biological OSMAC approaches. *Molecules* 26:3517. 10.3390/molecules26123517 34207697 PMC8228313

[B4] Bais H. P. Vepachedu R. Lawrence C. B. Stermitz F. R. Vivanco J. M. (2003). Molecular and biochemical characterization of an enzyme responsible for the formation of hypericin in St. John’s wort (*Hypericum perforatum* L.). *J. Biol. Chem.* 278 32413–32422. 10.1074/jbc.M301681200 12799379

[B5] Bauer A. Brönstrup M. Brönstrup B. (2014). Industrial natural product chemistry for drug discovery and development. *R. Soc. Chem.* 31:35. 10.1039/c3np70058e 24142193

[B6] Carrión V. J. Perez-Jaramillo J. Cordovez V. Tracanna V. de Hollander M. Ruiz-Buck D. (2019). Pathogen-induced activation of disease-suppressive functions in the endophytic root microbiome. *Science* 366 606–612. 10.1126/science.aaw9285 31672892

[B7] Castro-Moretti F. R. Gentzel I. N. Mackey D. A. Alonso A. P. (2020). Metabolomics as an emerging tool for the study of plant-pathogen interactions. *Metabolites* 10:52. 10.3390/metabo10020052 32013104 PMC7074241

[B8] Chithra S. Jasim B. Anisha C. Mathew J. Radhakrishnan E. K. (2014a). LC-MS/MS based identification of piperine production by endophytic *Mycosphaerella* sp. PF13 from *Piper nigrum*. *Appl. Biochem. Biotechnol.* 173 30–35. 10.1007/s12010-014-0832-3 24691878

[B9] Chithra S. Jasim B. Sachidanandan P. Jyothis M. Radhakrishnan E. K. (2014b). Piperine production by endophytic fungus *Colletotrichum gloeosporioides* isolated from *Piper nigrum*. *Phytomedicine* 21 534–540. 10.1016/j.phymed.2013.10.020 24268806

[B10] Cipriano M. A. P. Freitas-Iório R. d. P. Dimitrov M. R. de Andrade S. A. L. Kuramae E. E. Silveira A. P. D. (2021). Plant-growth endophytic bacteria improve nutrient use efficiency and modulate Foliar N-Metabolites in sugarcane seedling. *Microorganisms* 9:479. 10.3390/microorganisms9030479 33669086 PMC7996552

[B11] Correa-Galeote D. Bedmar E. J. Arone G. J. (2018). Maize endophytic bacterial diversity as affected by soil cultivation history. *Front. Microbiol.* 9:484. 10.3389/fmicb.2018.00484 29662471 PMC5890191

[B12] Dellafiora L. Galaverna G. Cruciani G. Dall’Asta C. Bruni R. (2018). On the mechanism of action of anti-inflammatory activity of hypericin: an in silico study pointing to the relevance of Janus kinases inhibition. *Molecules* 23:3058. 10.3390/molecules23123058 30467287 PMC6321526

[B13] Deng Y. Lu S. (2017). Biosynthesis and regulation of phenylpropanoids in plants. *Crit. Rev. Plant Sci.* 36 257–290. 10.1080/07352689.2017.1402852

[B14] Devari S. Jaglan S. Kumar M. Deshidi R. Guru S. Bhushan S. (2014). Capsaicin production by *Alternaria alternata*, an endophytic fungus from *Capsicum annum*; LC-ESI-MS/MS analysis. *Phytochemistry* 98 183–189. 10.1016/j.phytochem.2013.12.001 24378219

[B15] Eyberger A. L. Dondapati R. Porter J. R. (2006). Endophyte fungal isolates from *Podophyllum peltatum* produce podophyllotoxin. *J. Nat. Prod.* 69 1121–1124. 10.1021/np060174f 16933860

[B16] Fraser C. M. Chapple C. (2011). The phenylpropanoid pathway in *Arabidopsis*. *Arabidopsis Book* 9:152. 10.1199/tab.0152 22303276 PMC3268504

[B17] Gakuubi M. M. Munusamy M. Liang Z.-X. Ng S. B. (2021). Fungal endophytes: a promising frontier for discovery of novel bioactive compounds. *J. Fungi* 7:786. 10.3390/jof7100786 34682208 PMC8538612

[B18] García-Latorre C. Rodrigo S. Santamaría O. (2021). “Endophytes as plant nutrient uptake-promoter in plants,” in *Endophytes: Mineral Nutrient Management, Sustainable Development and Biodiversity*, Vol. 3 eds Maheshwari D. K. Dheeman S. (Cham: Springer). 10.1007/978-3-030-65447-4_11

[B19] Ghanbari-Movahed M. Kaceli T. Mondal A. Farzaei M. H. Bishayee A. (2021). Recent advances in improved anticancer efficacies of camptothecin nano-formulations: a Systematic Review. *Biomedicines* 9:480. 10.3390/biomedicines9050480 33925750 PMC8146681

[B20] Giauque H. Hawkes C. V. (2013). Climate affects symbiotic fungal endophyte diversity and performance. *Am. J. Bot.* 100 1435–1444. 10.3732/ajb.1200568 23813587

[B21] Gouda S. Das G. Sen S. K. Shin H. S. Patra J. K. (2016). Endophytes: a treasure house of bioactive compounds of medicinal importance. *Front. Microbiol.* 7:1538. 10.3389/fmicb.2016.01538 27746767 PMC5041141

[B22] Guevara-Araya M. J. Vilo C. Urzúa A. Gonzalez-Teuber M. (2020). Differences in community composition of endophytic fungi between above- and below-ground tissues of *Aristolochia chilensis* in an arid ecosystem. *Rev. Chil. Hist. Nat.* 93:3. 10.1186/s40693-020-00091-y

[B23] Guo L. Yu H. Wang B. Vescio K. Deiulio G. A. Yang H. (2021). Metatranscriptomic comparison of endophytic and pathogenic fusarium-*Arabidopsis* interactions reveals plant transcriptional plasticity. *Mol. Plant Microbe Interact.* 34 1071–1083. 10.1094/MPMI-03-21-0063-R 33856230 PMC9048145

[B24] Harrison J. G. Griffin E. A. (2020). The diversity and distribution of endophytes across biomes, plant phylogeny and host tissues: how far have we come and where do we go from here? *Environ. Microbiol.* 22 2107–2123. 10.1111/1462-2920.14968 32115818 PMC7679042

[B25] Hayden H. L. Rochfort S. J. Ezernieks V. Savin K. W. Mele P. M. (2019). Metabolomics approaches for the discrimination of disease suppressive soils for *Rhizoctonia solani* AG8 in cereal crops using 1H NMR and LC-MS. *Sci. Total Environ.* 651 1627–1638. 10.1016/j.scitotenv.2018.09.249 30360288

[B26] Hosseyni-Moghaddam M. S. Soltani J. (2014). Bioactivity of endophytic Trichoderma fungal species from the plant family Cupressaceae. *Ann. Microbiol.* 64 753–761. 10.1007/s13213-013-0710-1

[B27] Huang J. X. Zhang J. Zhang X. R. Zhang K. Zhang X. He X. R. (2014). *Mucor fragilis* as a novel source of the key pharmaceutical agents podophyllotoxin and kaempferol. *Pharm Biol.* 52 1237–1243. 10.3109/13880209.2014.885061 24863281

[B28] Huang W. Sun D. Chen L. An Y. (2021). Integrative analysis of the microbiome and metabolome in understanding the causes of sugarcane bitterness. *Sci. Rep.* 11:6024. 10.1038/s41598-021-85433-w 33727648 PMC7966368

[B29] Ibrahim A. Tanney J. B. Fei F. Seifert K. A. Christopher Cutler G. Capretta A. (2020). Metabolomic-guided discovery of cyclic nonribosomal peptides from *Xylaria ellisii* sp. nov., a leaf and stem endophyte of *Vaccinium angustifolium*. *Sci. Rep.* 10:4599. 10.1038/s41598-020-61088-x 32165688 PMC7067778

[B30] Johnston-Monje D. Raizada M. N. (2011). “Integration of biotechnologies | plant and endophyte relationships: nutrient management,” in *Comprehensive Biotechnology*, 2nd Edn, Vol. 4 ed. Moo-Young M. (Amsterdam: Elsevier), 713–727. 10.1016/B978-0-08-088504-9.00264-6

[B31] Kaul S. Sharma T. Dhar M. K. (2016). “Omics” tools for better understanding the plant–endophyte interactions. *Front. Plant Sci.* 7:955. 10.3389/fpls.2016.00955 27446181 PMC4925718

[B32] Kilani-Morakchi S. Morakchi-Goudjil H. Sifi K. (2021). Azadirachtin-based insecticide: Overview, risk assessments, and future directions. *Front. Agron.* 3:32. 10.3389/fagro.2021.676208

[B33] Krings M. Taylor T. N. Hass H. Kerp H. Dotzler N. Hermsen E. J. (2007). Fungal endophytes in a 400-million-yr-old land plant: infection pathways, spatial distribution, and host responses. *N. Phytol.* 174 648–657. 10.1111/j.1469-8137.2007.02008.x 17447919

[B34] Kumar P. Singh B. Thakur V. Thakur A. Thakur N. Pandey D. (2019). Hyper-production of taxol from *Aspergillus fumigatus*, an endophytic fungus isolated from Taxus sp. of the Northern Himalayan region. *Biotechnol. Rep.* 24:e00395. 10.1016/j.btre.2019.e00395 31799144 PMC6881681

[B35] Kusari S. Lamshoö M. Kusari P. Gottfried S. Zuü S. Louven K. (2014a). Endophytes are hidden producers of maytansine in *Putterlickia* roots. *J. Nat. Prod.* 77 2577–2584. 10.1021/np500219a 25478947

[B36] Kusari S. Singh S. Jayabaskaran C. (2014b). Rethinking production of Taxol§(paclitaxel) using endophyte biotechnology. *Trends Biotechnol.* 32 304–311. 10.1016/j.tibtech.2014.03.011 24810040

[B37] Kusari S. Lamshöft M. Zühlke S. Spiteller M. (2008). An Endophytic fungus from *Hypericum perforatum* that produces hypericin. *J. Nat. Prod.* 71 159–162. 10.1021/np070669k 18220354

[B38] Kusari S. Verma V. C. Lamshoeft M. Spiteller M. (2012). An endophytic fungus from *Azadirachta indica* A. Juss. that produces azadirachtin. *World J. Microb. Biotechnol.* 28 1287–1294. 10.1007/s11274-011-0876-2 22805849

[B39] Kusari S. Zühlke S. Spiteller M. (2009). An endophytic Fungus from *Camptotheca acuminata* that produces camptothecin and analogues. *J. Nat. Prod.* 72 2–7. 10.1021/np800455b 19119919

[B40] Liao H.-L. Bonito G. Rojas J. A. Hameed K. Wu S. Schadt C. W. (2019). Fungal endophytes of *Populus trichocarpa* alter host phenotype, gene expression, and rhizobiome composition. *Mol. Plant Microbe Interact.* 32 853–864. 10.1094/MPMI-05-18-0133-R 30699306

[B41] Llorens E. Sharon O. Camañes G. García-Agustín P. Sharon A. (2019). Endophytes from wild cereals protect wheat plants from drought by alteration of physiological responses of the plants to water stress. *Environ. Microbiol.* 21 3299–3312. 10.1111/1462-2920.14530 30637909

[B42] Lu Y. Che J. Xu X. Pang B. Zhao X. Liu Y. (2020). Metabolomics reveals the response of the phenylpropanoid biosynthesis pathway to starvation treatment in the grape endophyte *Alternaria* sp. MG1. *J. Agricult. Food Chem.* 68 1126–1135. 10.1021/acs.jafc.9b05302 31891261

[B43] Maciá-Vicente J. G. Shi Y. N. Cheikh-Ali Z. Grün P. Glynou K. Kia S. H. (2018). Metabolomics-based chemotaxonomy of root endophytic fungi for natural products discovery. *Environ. Microbiol.* 20 1253–1270. 10.1111/1462-2920.14072 29441701

[B44] Mendes R. Kruijt M. Bruijn I. de Dekkers E. Voort M. (2011). Deciphering the rhizosphere microbiome for disease-suppressive Bacteria. *Science* 332 1093–1097. 10.1126/science.1202007 21551032

[B45] Mintoo M. N. Mishra S. Prem D. K. (2019). Isolation and characterization of endophytic bacteria from *Piper longum*. *Proc. Natl. Acad. Sci. India Sect. B Biol. Sci.* 89 1447–1454. 10.1007/s40011-018-01064-8

[B46] Mir R. A. Kaushik P. S. Chowdery R. A. Anuradha M. (2015). Elicitation of forskolin in cultures of rhizactonia bataticola-a phytochemical synthesizing endophytic fungi. *Int. J. Pharm. Pharmaceut. Sci.* 7 185–189.

[B47] Mishra S. Bhattacharjee A. Sharma S. (2021a). An ecological insight into the multifaceted world of plant-endophyte Association. *Crit. Rev. Plant Sci.* 40 127–146. 10.1080/07352689.2021.1901044

[B48] Mishra S. Sahu P. K. Agarwal V. Singh N. (2021c). Exploiting endophytic microbes as micro-factories for plant secondary metabolite production. *Appl. Microbiol. Biotechnol.* 105 6579–6596. 10.1007/s00253-021-11527-0 34463800

[B49] Mishra S. Goyal D. Phurailatpam L. (2021b). Targeted 16S rRNA gene and ITS2 amplicon sequencing of leaf and spike tissues of *Piper longum* identifies new candidates for bioprospecting of bioactive compounds. *Arch. Microbiol.* 203 3851–3867. 10.1007/s00203-021-02356-w 34013420

[B50] Mohamed E. M. Hetta M. H. Rateb M. E. Selim M. A. Aboulmagd A. M. Badria F. A. (2020). Bioassay-guided isolation, metabolic profiling, and docking studies of hyaluronidase inhibitors from *Ravenala madagascariensis*. *Molecules* 25:1714. 10.3390/molecules25071714 32276509 PMC7180949

[B51] Mohamad O. A. A. Li L. Ma J. B. Hatab S. Xu L. Gua J. W. (2018). Evaluation of the antimicrobial activity of endophytic bacterial populations from Chinese traditional medicinal plant licorice and characterization of the bioactive secondary metabolites produced by Bacillus atrophaeus against *Verticillium dahlia*. *Front. Microbiol.* 9:924. 10.3389/fmicb.2018.00924 29867835 PMC5954123

[B52] Mohinudeen K. I. A. H. Pandey S. Kanniyappan H. Muthuvijayan V. Srivastava S. (2021). Screening and selection of camptothecin producing endophytes from *Nothapodytes nimmoniana*. *Sci. Rep.* 11:11205. 10.1038/s41598-021-90778-3 34045605 PMC8159990

[B53] Na R. Jiajia L. Dongliang Y. Yingzi P. Juan H. Xiong L. (2016). Indentification of vincamine indole alkaloids producing endophytic fungi isolated from *Nerium indicum*, Apocynaceae. *Microbiol. Res.* 192 114–121. 10.1016/j.micres.2016.06.008 27664729

[B54] Nalbantoglu S. (2019). “Metabolomics: basic principles and strategies,” in *Molecular Medicine*, eds Nalbantoglu S. Amri H. (London: IntechOpen), 137–150. 10.5772/intechopen.88563

[B55] Nischitha R. Shivanna M. B. (2021). Metabolite fingerprinting, in vitro antimicrobial and antioxidant activities and in-silico docking in *Alloteropsis cimicina* and its endophytic fungus *Penicillium pinophilum*. *Mol. Biol. Rep.* 48 4021–4037. 10.1007/s11033-021-06410-0 34023986

[B56] Oita S. Ibáñez A. Lutzoni F. Miadlikowska J. Geml J. Lewis L. A. (2021). Climate and seasonality drive the richness and composition of tropical fungal endophytes at a landscape scale. *Commun. Biol.* 4:313. 10.1038/s42003-021-01826-7 33750915 PMC7943826

[B57] Pandey S. S. Singh S. Pandey H. Srivastava M. Ray T. Soni S. (2018). Endophytes of *Withania somnifera* modulate in planta content and the site of withanolide biosynthesis. *Sci. Rep.* 8:5450. 10.1038/s41598-018-23716-5 29615668 PMC5882813

[B58] Porzel A. Farag M. A. Mülbradt J. Wessjohann L. A. (2014). Metabolite profiling and fingerprinting of *Hypericum* species: a comparison of MS and NMR metabolomics. *Metabolomics* 10 574–588. 10.1007/s11306-013-0609-7

[B59] Qiu M. Xie R. S. Shi Y. Zhang H. Chen H. M. (2010). Isolation and identification of two flavonoid-producing endophytic fungi from *Ginkgo biloba* L. *Ann. Microbiol.* 60 143–150. 10.1007/s13213-010-0016-5

[B60] Rai N. Keshri P. K. Verma A. Kamble S. C. Mishra P. Barik S. (2021). Plant associated fungal endophytes as a source of natural bioactive compounds. *Mycology* 12 139–159. 10.1080/21501203.2020.1870579 34552808 PMC8451683

[B61] Ran X. Zhang G. Li S. Wang J. (2017). Characterization and antitumor activity of camptothecin from endophytic fungus Fusarium solani isolated from *Camptotheca acuminate*. *Afr. Health Sci.* 17 566–574. 10.4314/ahs.v17i2.34 29062355 PMC5637045

[B62] Rigobelo E. C. Baron N. C. (2021). Endophytic fungi: a tool for plant growth promotion and sustainable agriculture. *Mycology* 10.1080/21501203.2021.1945699 35186412 PMC8856089

[B63] Salehi B. Mishra A. P. Nigam M. Sener B. Kilic M. Sharifi-Rad M. (2018). Resveratrol: A double-edged sword in health benefits. *Biomedicines* 6:91. 10.3390/biomedicines6030091 30205595 PMC6164842

[B64] Sathiyabama M. Parthasarathy R. (2018). Withanolide production by fungal endophyte isolated from *Withania somnifera*. *Nat. Prod. Res.* 32 1573–1577. 10.1080/14786419.2017.1389934 29034745

[B65] Schafhauser T. Jahn L. Kirchner N. Kulik A. Flor L. Lang A. (2019). Antitumor astins originate from the fungal endophyte *Cyanodermella asteris* living within the medicinal plant *Aster tataricus*. *Proc. Natl. Acad. Sci. U.S.A.* 116 26909–26917. 10.1073/pnas.1910527116/-/DCSupplemental 31811021 PMC6936678

[B66] Scherling C. Ulrich K. Ewald D. Weckwerth W. (2009). A metabolic signature of the beneficial interaction of the endophyte *Paenibacillus* sp. isolate and in vitro-grown poplar plants revealed by metabolomics. *Mol. Plant Microbe Interact. MPMI* 22 1032–1037. 19589078 10.1094/MPMI-22-8-1032

[B67] Sebola T. E. Uche-Okereafor N. C. Mekuto L. Makatini M. M. Green E. Mavumengwana V. (2020). Antibacterial and anticancer activity and untargeted secondary metabolite profiling of crude bacterial endophyte extracts from *Crinum macowanii* Baker leaves. *Int. J. Microbiol.* 2020:8839490. 10.1155/2020/8839490 33488726 PMC7803143

[B68] Segers K. Declerck S. Mangelings D. Heyden Y. van der Eeckhaut A. (2019). Analytical techniques for metabolomic studies: a review. *Bioanalysis* 11 2297–2318. 10.4155/bio-2019-0014 31845604

[B69] Sessitsch A. Hardoim P. Döring J. Weilharter A. Krause A. Woyke T. (2012). Functional characteristics of an endophyte community colonizing rice roots as revealed by metagenomic analysis. *Mol. Plant Microbe Interact. MPMI* 25 28–36. 21970692 10.1094/MPMI-08-11-0204

[B70] Shulaev V. Cortes D. Miller G. Mittler R. (2008). Metabolomics for plant stress response. *Physiol. Plant.* 132 199–208. 10.1111/j.1399-3054.2007.01025.x 18251861

[B71] Shweta S. Zuehlke S. Ramesha B. T. Priti V. Mohana Kumar P. Ravikanth G. (2010). Endophytic fungal strains of *Fusarium solani*, from *Apodytes dimidiata* E. Mey. ex Arn (Icacinaceae) produce camptothecin, 10-hydroxycamptothecin and 9-methoxycamptothecin. *Phytochemistry* 71 117–122. 10.1016/j.phytochem.2009.09.030 19863979

[B72] Spjut R. W. Cassady J. M. Mccloud T. Suffness M. Norris D. H. Cragg G. M. (1988). Variation in cytotoxicity and antitumor activity among samples of the moss *Claopodium crispifolium* (Thuidiaceae). *Econ. Bot.* 42 62–72.

[B73] Stierle A. Strobel G. Stierle D. (1993). Taxol and taxane production by *Taxomyces andreanae*, an endophytic fungus of Pacific yew. *Science* 260 214–216. 10.1126/science.8097061 8097061

[B74] Tawfike A. F. Romli M. Clements C. Abbott G. Young L. Schumacher M. (2019). Isolation of anticancer and anti-trypanosome secondary metabolites from the endophytic fungus *Aspergillus flocculus* via bioactivity guided isolation and MS based metabolomics. *J. Chromatogr. B Anal. Technol. Biomed. Life Sci.* 1106–1107 71–83. 10.1016/j.jchromb.2018.12.032 30658264

[B75] Tawfike A. F. Tate R. Abbott G. Young L. Viegelmann C. Schumacher M. (2017). Metabolomic tools to assess the chemistry and bioactivity of endophytic *Aspergillus* strain. *Chem. Biodiver.* 14:40. 10.1002/cbdv.201700040 28672096

[B76] Tebani A. Afonso C. Bekri S. (2018). Advances in metabolome information retrieval: turning chemistry into biology. Part I: analytical chemistry of the metabolome. *J. Inherit. Metab. Dis.* 41 379–391. 10.1007/s10545-017-0074-y 28840392 PMC5959978

[B77] Tiwari A. Mahadik K. R. Gabhe S. Y. (2020). Piperine: acomprehensive review of method of isolation, purification, and biological properties. *Med. Drug Discov.* 7:100027. 10.1016/j.medidd.2020.100027

[B78] Tiwari P. Bae H. (2020). Horizontal gene transfer and endophytes: An Implication for the acquisition of novel traits. *Plants* 9:305. 10.3390/plants9030305 32121565 PMC7154830

[B79] Toghueo R. M. K. Sahal D. Boyom F. F. (2020). Recent advances in inducing endophytic fungal specialized metabolites using small molecule elicitors including epigenetic modifiers. *Phytochemistry* 174:112338. 10.1016/j.phytochem.2020.112338 32179305

[B80] Verma S. K. Sahu P. K. Kumar K. Pal G. Gond S. K. Kharwar R. N. (2021). Endophyte roles in nutrient acquisition, root system architecture development and oxidative stress tolerance. *J. Appl. Microbiol.* 131 2161–2177. 10.1111/jam.15111 33893707

[B81] Verma V. C. Lobkovsky E. Gange A. C. Singh S. K. Prakash S. (2011). Piperine production by endophytic fungus *Periconia* sp. isolated from *Piper longum* L. *J. Antib.* 64 427–431. 10.1038/ja.2011.27 21505472

[B82] Vigneshwari A. Vid Rakk D. Németh A. Ndor Kocsubé S. Mi Kiss N. Csupor D. (2019). Host metabolite producing endophytic fungi isolated from *Hypericum perforatum*. *PLoS One* 14:60. 10.1371/journal.pone.0217060 31112560 PMC6529008

[B83] Wang L. Ren L. Li C. Gao C. Liu X. Wang M. (2019). Effects of endophytic fungi diversity in different coniferous species on the colonization of *Sirex noctilio* (Hymenoptera: Siricidae). *Sci. Rep.* 9:5077. 10.1038/s41598-019-41419-3 30911076 PMC6433867

[B84] White J. F. Kingsley K. L. Zhang Q. Verma R. Obi N. Dvinskikh S. (2021). Review: Endophytic microbes and their potential applications in crop management. *Pest Manag. Sci.* 75 2558–2565. 10.1002/ps.5527 31228333 PMC6771842

[B85] White P. T. Subramanian C. Motiwala H. F. Cohen M. S. (2016). Natural withanolides in the treatment of chronic diseases. *Adv. Exp. Med. Biol.* 928 329–373. 10.1007/978-3-319-41334-1_14 27671823 PMC7121644

[B86] Yamamoto N. Takano T. Tanaka K. Ishige T. Terashima S. Endo C. (2015). Comprehensive analysis of transcriptome response to salinity stress in the halophytic turf grass *Sporobolus virginicus*. *Front. Plant Sci.* 6:241. 10.3389/fpls.2015.00241 25954282 PMC4404951

[B87] Yadav V. Krishnan A. Vohora D. (2020). A systematic review on *Piper longum* L.: bridging traditional knowledge and pharmacological evidence for future translational research. *J. Ethnopharmacol.* 247:112255. 10.1016/j.jep.2019.112255 31568819

[B88] Zimmerman N. B. Vitousek P. M. (2012). Fungal endophyte communities reflect environmental structuring across a Hawaiian landscape. *Proc. Natl. Acad. Sci. U.S.A.* 109 13022–13027. 10.1073/pnas.1209872109 22837398 PMC3420199

