# Supplementary material for: Metabolomic Insights Into Endophyte-Derived Bioactive Compounds
Source: Front Microbiol. 2022 Mar 2;13:835931. doi: 10.3389/fmicb.2022.835931 (PMC8926391; doi:10.3389/fmicb.2022.835931)
Supplement: Supplementary file 1 [file Table_1.DOCX]

**Supplementary Table 1**: Details of various classes of endophyte-derived bioactive compounds

| **Compound(s)** | **Function(s)** | **Endophyte(s)** | **Host plant** | **Reference** |
| --- | --- | --- | --- | --- |
| **ALKALOIDS** | | | | |
| Cytochalasin D | Antifungal | *Xylaria* sp*.* | *Paullinia cupana* | Elias et al., 2018 |
| Paclitaxel | Anticancer | *Aspergillus fumigatus* | *Taxus* sp. | Kumar et al., 2019 |
| Vincamine | Cardiovascular and cerebrovascular protective | *Geomyces* sp. | *Nerium indicum* | Na et al., 2016 |
| Camptothecin | Antitumour | *Fusarium solani* | *Camptotheca acuminata* | Ran et al., 2017 |
| 3β,5α-dihydroxy-6β-acetoxy-ergosta-7,22-diene | Antibacterial | *Colletotrichum* sp. | *Artemisia annua* | Sharma et al., 2021 |
| Piperine | Antimicrobial | *Colletotrichum gloeosporioides* | *Piper nigrum* | White et al., 2016 |
|  | | | | |
| **ANTIBIOTICS** | | | | |
| Actinomycin D and actinomycin X0β | Antibacterial and antitumor | *Streptomyces parvulus* | *Aloe barbadensis miller* | Chandrakar and Gupta, 2019 |
| Surfactin, Iturin, and Fengycin | Antifungal, antibacterial | *Bacillus*sp. | *Bacopa monnieri* | Jasim et al., 2016 |
| Munumbicins | Wide spectrum of activity against plant pathogenic fungi and bacteria | *Streptomyces coelicolor* | *Kennedia nigricans* | Golinska et al., 2015 |
| Clethramycin | Antifungal | *Streptomyces hygroscopicus* | *Pteridium aquilinum* | Golinska et al., 2015 |
| Putative antibiotic | Antibacterial | *Pantoea* sp. | *Solanum mauritianum* | Uche-Okereafor et al., 2019 |
| Hypericin  emodin | Antibacterial | *Epicoccum nigrum* | *Hypericum perforatum* | Vigneshwari et al., 2019 |
| Fusolanone B | Antibacterial | *Fusarium solani* | *Rhizophora apiculata* | Zhou et al., 2019 |
|  | | | | |
| **FLAVONOIDS** | | | | |
| Kaempferol | Antioxidant | *Eupenicillium parvum* | *Inopodophyllum hexandrum* | Huang et al., 2014 |
| Rutin | Antioxidant | *Fusarium* sp. | *Fritillaria unibracteata* | Pan et al., 2017 |
|  | | | | |
| **PHENOLICS** | | | | |
| Salidroside and p-tyrosol | Antioxidant | *Phialocephala fortinii* | *Rhodiola rose* | Cui et al., 2016 |
| Orcinol | Antioxidant | *Aspergillus nidulans var. dentatus*LMA 1705 | *Passiflora incarnata* | da Silva et al., 2020 |
| Altenusin | Antifungal | *Alternaria alternata* | *Terminalia chebula* | Phaopongthai et al., 2013 |
| Gallic acid | Antibacterial and antioxidant | *Bacillus* sp. strain CPS003 | *Oryza sativa* L. var. indica cv.RD41 | Rangjaroen et al., 2019 |
| Graphislactone A | Antioxidant and free radical scavenger | *Cephalosporium* sp. | *Trachelospermum jasminoides* | Song et al., 2005 |
|  | | | | |
| **PHYTOHORMONES** | | | | |
| IAA | Phosphate and potassium solubilization, antifungal activity | *Rhizobium* sp*.* R5 | *Oryza sativa* | Aye Khai et al., 2016 |
| IAA, Gibberelic acid (GA) | Plant growth promotion | *Microbacterium laevaniformans* RS0111, *Bacillus tequilensis* RHS01 | *Oryza sativa*  *Oryza rufipogon* | Borah et al., 2021 |
| IAA, GA, siderophore | Plant growth promotion, oxidative stress responses | *Penicillium citrinum* LWL4 *Aspergillus terreus* LWL5 | *Helianthus annuus* | Nayak et al., 2020 |
| Indole acetic acid (IAA) | Plant growth promotion, phosphate solubilization, nitrogen fixation | *Bacillus megaterium, Lelliottia amnigena, Pantoea agglomerans and Alcaligenes*sp. | *Pairs polyphylla var. yunnanensis* | Tao et al., 2022 |
|  | | | | |
| **QUINONES** | | | | |
| Torreyanic acid | Cytotoxic agent | *Pestalotiopsis microsporum* | *Torreya taxifolia* | Kaul et al., 2012 |
| Fumiquinone B | Fungicides | *Neopestalotiopsis* sp. | Begonia sp. | Grigoletto et al., 2019 |
| Brefeldin A | Antiviral | Nigrospora sp. YE3033 | Aconitum carmichaeli | Zhang et al., 2016 |
|  | | | | |
| **STEROIDS** | | | | |
| Stigmasterol | Anti-osteoarthritic, anti-hypercholesterolemia, antioxidant, anti-inflammatory | *Aspergillus fumigatus* | *Ocimum basilicum* | Kaur et al., 2014 |
| Ergosta-7,9(14),22-triene-3β-ol | Antifungal | *Nigrosspora sphaerica* | *Vinca rosea* | Metwaly et al., 2014 |
|  | | | | |
| **TERPENOIDS** | | | | |
| 14-nordrimane sesquiterpenoid | Antiviral | Phoma sp. | Aconitum vilmorinianum | Liu et al., 2019 |
| Viridiflorol | Antifungal | *Serendipita indica* | *Solanum lycopersicum* | Ntana et al., 2021 |
| Vindoline, Vinblastine, and Vincristine | Anticancer | *Choanephora infundibulifera, Paenibacillus* sp*., Curvularia* sp*.* | *Catharanthus roseus* | Singh et al., 2021 |

**References**

Aye Khai, A., San Yu, S., May Sev, T., and Aung, A. (2016). Evaluation of endophytic bacteria from some rice varieties for plant growth promoting activities. *J. Sci. Innov. Res.* 5, 144–148. Available at: www.jsirjournal.com.

Borah, M., Das, S., Bora, S. S., Boro, R. C., and Barooah, M. (2021). Comparative assessment of multi-trait plant growth-promoting endophytes associated with cultivated and wild Oryza germplasm of Assam, India. *Arch. Microbiol.* 203, 2007–2028. doi:10.1007/s00203-020-02153-x.

Chandrakar, S., and Gupta, A. K. (2019). Actinomycin-producing endophytic *Streptomyces parvulus* associated with root of *Aloe vera* and optimization of conditions for antibiotic production. *Probiotics Antimicrob. Proteins* 11, 1055–1069. doi:10.1007/s12602-018-9451-6.

Cui, J., Guo, T., Chao, J., Wang, M., and Wang, J. (2016). Potential of the endophytic fungus *Phialocephala fortinii* Rac56 found in *Rhodiola* plants to produce salidroside and p-tyrosol. *Molecules* 21. doi:10.3390/molecules21040502.

da Silva, M. H. R., Cueva-Yesquén, L. G., Júnior, S. B., Garcia, V. L., Sartoratto, A., de Angelis, D. de F., et al. (2020). Endophytic fungi from *Passiflora incarnata*: an antioxidant compound source. *Arch. Microbiol.* 202, 2779–2789. doi:10.1007/s00203-020-02001-y.

Elias, L. M., Fortkamp, D., Sartori, S. B., Ferreira, M. C., Gomes, L. H., Azevedo, J. L., et al. (2018). The potential of compounds isolated from *Xylaria* spp. as antifungal agents against anthracnose. *Brazilian J. Microbiol.* 49, 840–847. doi:10.1016/j.bjm.2018.03.003.

Golinska, P., Wypij, M., Agarkar, G., Rathod, D., Dahm, H., and Rai, M. (2015). Endophytic actinobacteria of medicinal plants: Diversity and bioactivity. *Antonie van Leeuwenhoek, Int. J. Gen. Mol. Microbiol.* 108, 267–289. doi:10.1007/s10482-015-0502-7.

Grigoletto, D. F., Correia, A. M. L., Abraham, W. R., Rodrigues, A., Assis, M. A., Ferreira, A. G., et al. (2019). Secondary metabolites produced by endophytic fungi: Novel antifungal activity of fumiquinone B. *Acta Sci. - Biol. Sci.* 41. doi:10.4025/actascibiolsci.v41i1.48785.

Huang, J. X., Zhang, J., Zhang, X. R., Zhang, K., Zhang, X., and He, X. R. (2014). Mucor fragilis as a novel source of the key pharmaceutical agents podophyllotoxin and kaempferol. *Pharm. Biol.* 52, 1237–1243. doi:10.3109/13880209.2014.885061.

Jasim, B., Sreelakshmi, K. S., Mathew, J., and Radhakrishnan, E. K. (2016). Surfactin, iturin, and fengycin biosynthesis by endophytic *Bacillus* sp. from *Bacopa monnieri*. *Microb. Ecol.* 72, 106–119. doi:10.1007/s00248-016-0753-5.

Kaul, S., Gupta, S., Ahmed, M., and Dhar, M. K. (2012). Endophytic fungi from medicinal plants: A treasure hunt for bioactive metabolites. *Phytochem. Rev.* 11, 487–505. doi:10.1007/s11101-012-9260-6.

Kaur, N., Chaudhary, J., Jian, A., and Kishore, L. (2014). Stigmasterol: A comprehensive Review. 5, 4275–4280. doi:10.13040/IJPSR.0975-8232.5(10).4275-80.

Kumar, P., Singh, B., Thakur, V., Thakur, A., Thakur, N., Pandey, D., et al. (2019). Hyper-production of taxol from *Aspergillus fumigatus*, an endophytic fungus isolated from *Taxus* sp. of the Northern Himalayan region. *Biotechnol. Reports* 24, e00395. doi:10.1016/j.btre.2019.e00395.

Liu, S. S., Jiang, J. X., Huang, R., Wang, Y. T., Jiang, B. G., Zheng, K. X., et al. (2019). A new antiviral 14-nordrimane sesquiterpenoid from an endophytic fungus *Phoma* sp. *Phytochem. Lett.* 29, 75–78. doi:10.1016/j.phytol.2018.11.005.

Metwaly, A. M., Kadry, H. A., El-Hela, A. a., Mohammad, A.-E. I., Ma, G., Cutler, S. J., et al. (2014). Nigrosphaerin A a new isochromene derivative from the endophytic fungus *Nigrospora sphaerica*. *Phytochem. Lett.* 17, 1–5. doi:10.1016/j.phytol.2013.09.001.

Na, R., Jiajia, L., Dongliang, Y., Yingzi, P., Juan, H., Xiong, L., et al. (2016). Indentification of vincamine indole alkaloids producing endophytic fungi isolated from *Nerium indicum*, Apocynaceae. *Microbiol. Res.* 192, 114–121. doi:10.1016/j.micres.2016.06.008.

Nayak, S., Samanta, S., and Mukherjee, A. K. (2020). Beneficial role of *Aspergillus* sp. in agricultural soil and environment. *Front. Soil Environ. Microbiol.*, 17–36. doi:10.1201/9780429485794-3.

Ntana, F., Bhat, W. W., Johnson, S. R., Jørgensen, H. J. L., Collinge, D. B., Jensen, B., et al. (2021). A sesquiterpene synthase from the endophytic fungus *Serendipita indica* catalyzes formation of viridiflorol. *Biomolecules* 11, 1–16. doi:10.3390/biom11060898.

Pan, F., Su, T. J., Cai, S. M., and Wu, W. (2017). Fungal endophyte-derived *Fritillaria unibracteata* var. wabuensis: Diversity, antioxidant capacities in vitro and relations to phenolic, flavonoid or saponin compounds. *Sci. Rep.* 7, 1–14. doi:10.1038/srep42008.

Phaopongthai, J., Wiyakrutta, S., Meksuriyen, D., Sriubolmas, N., and Suwanborirux, K. (2013). Azole-synergistic anti-candidal activity of altenusin, a biphenyl metabolite of the endophytic fungus *Alternaria alternata* isolated from *Terminalia chebula* Retz. *J. Microbiol.* 51, 821–828. doi:10.1007/s12275-013-3189-3.

Ran, X., Zhang, G., Li, S., and Wang, J. (2017). Characterization and antitumor activity of camptothecin from endophytic fungus *Fusarium solani* isolated from *Camptotheca acuminate*. *Afr. Health Sci.* 17, 566–574. doi:10.4314/ahs.v17i2.34.

Rangjaroen, C., Lumyong, S., Sloan, W. T., and Sungthong, R. (2019). Herbicide-Tolerant endophytic bacteria of rice plants as the biopriming agents for fertility recovery and disease suppression of unhealthy rice seeds. *BMC Plant Biol.* 19, 1–16. doi:10.1186/s12870-019-2206-z.

Sharma, H., Rai, A. K., Dahiya, D., Chettri, R., and Nigam, P. S. (2021). Exploring endophytes for in vitro synthesis of bioactive compounds similar to metabolites produced in vivo by host plants. *AIMS Microbiol.* 7, 175–199. doi:10.3934/MICROBIOL.2021012.

Singh, S., Pandey, S. S., Tiwari, R., Pandey, A., Shanker, K., and Kalra, A. (2021). Endophytic consortium with growth-promoting and alkaloid enhancing capabilities enhance key terpenoid indole alkaloids of *Catharanthus roseus* in the winter and summer seasons. *Ind. Crops Prod.* 166, 113437. doi:10.1016/j.indcrop.2021.113437.

Song, Y. C., Huang, W. Y., Sun, C., Wang, F. W., and Tan, R. X. (2005). Characterization of graphislactone A as the antioxidant and free radical-scavenging substance from the culture of *Cephalosporium* sp. IFB-E001, an endophytic fungus in Trachelospermum jasminoides. *Biol. Pharm. Bull.* 28, 506–509. doi:10.1248/bpb.28.506.

Tao, L., Qiuhong, L., Fuqiang, Y., Shuhui, Z., Suohui, T., and Linyuan, F. (2022). Plant growth-promoting activities of bacterial endophytes isolated from the medicinal plant *Pairs polyphylla* var. yunnanensis. *World J. Microbiol. Biotechnol.* 38, 1–10. doi:10.1007/s11274-021-03194-0.

Uche-Okereafor, N., Sebola, T., Tapfuma, K., Mekuto, L., Green, E., and Mavumengwana, V. (2019). Antibacterial activities of crude secondary metabolite extracts from pantoea species obtained from the stem of *Solanum mauritianum* and their effects on two cancer cell lines. *Int. J. Environ. Res. Public Health* 16. doi:10.3390/ijerph16040602.

Vigneshwari, A., Rakk, D., Németh, A., Kocsubé, S., Kiss, N., Csupor, D., et al. (2019). Host metabolite producing endophytic fungi isolated from *Hypericum perforatum*. *PLoS One* 14, 1–16. doi:10.1371/journal.pone.0217060.

White, P. T., Subramanian, C., Motiwala, H. F., and Cohen, M. S. (2016). Natural withanolides in the treatment of chronic diseases, in *Anti-inflammatory Nutraceutical and Chronic Diseases, Advances in Experimental Medicine and Biology*, ed. S. . et al. (eds. . Gupta (Springer International Publishing, Switzerland), 928. doi:10.1007/978-3-319-41334-1_14.

Zhang, S. P., Huang, R., Li, F. F., Wei, H. X., Fang, X. W., Xie, X. S., et al. (2016). Antiviral anthraquinones and azaphilones produced by an endophytic fungus *Nigrospora* sp. from Aconitum carmichaeli. *Fitoterapia* 112, 85–89. doi:10.1016/j.fitote.2016.05.013.

Zhou, G., Qiao, L., Zhang, X., Sun, C., Che, Q., Zhang, G., et al. (2019). Fusaricates H-K and fusolanones A-B from a mangrove endophytic fungus *Fusarium solani* HDN15-410. *Phytochemistry* 158, 13–19. doi:10.1016/j.phytochem.2018.10.035.
